# Dominance of P/Q-type calcium channels in depolarization-induced presynaptic fm dye release in cultured hippocampal neurons^☆^

**DOI:** 10.1016/j.neuroscience.2013.08.052

**Published:** 2013-12-03

**Authors:** B. Nimmervoll, B.E. Flucher, G.J. Obermair

**Affiliations:** Division of Physiology, Medical University Innsbruck, Fritz-Pregl-Str. 3, 6020 Innsbruck, Austria

**Keywords:** A, release amplitude, Aga, ω-agatoxin-IVA, Ca_V_, voltage-gated Ca^2+^ channel, CTx, ω-conotoxin GVIA, DIV, days in vitro, [K^+^], extracellular potassium concentration, *R*_f_, fractional release, *τ*, release time constant, voltage-gated Ca^2+^ channels, synapse function, N-type, P/Q-type, neurotransmitter release, calcium channel physiology

## Abstract

Neurotransmitter release probability is related by high power to the local concentration of calcium in presynaptic terminals, which in turn is controlled by voltage-gated calcium channels. P/Q- and N-type channels trigger synaptic transmission in the majority of neurons of the central nervous system. However, whether and under which conditions both channel types act cooperatively or independently is still insufficiently understood. Previous studies suggested either a dominance of N- or P/Q-type channels, or a synergistic action of both channels, depending on the experimental paradigms. Thus, to provide insight into the properties of neurotransmitter release in cultured mouse hippocampal neurons, we used quantitative analysis of FM dye release from presynaptic boutons induced by high potassium membrane depolarization. Increasing extracellular potassium concentrations revealed a sigmoid dependence of FM dye release to the stimulation strength. Individual and combined application of the P/Q- and N-type channel-specific blockers ω-agatoxin-IVA and ω-conotoxin-GVIA, respectively, allowed us to specifically isolate the contribution of both channel types to release triggered with 40 mM KCl. Analysis of the release kinetics and the fractional release amplitude demonstrate that, whereas in only 15% of the synapses release depended exclusively on P/Q-type channels, the majority of synapses (85%) contained both N- and P/Q-type channels. Nevertheless, the kinetics of FM dye release in synapses containing both channel types was determined by the P/Q-type channels. Together, our data suggest a more direct coupling of P/Q-type channels to synaptic release compared to N-type channels, which may explain the high prevalence of neurological P/Q-type channelopathies.

## Highlights

• We analyzed depolarization-induced synaptic FM dye release in hippocampal neurons.
• We pharmacologically isolated the contribution of voltage-gated Ca^2+^ channels.
• 85% of synapses utilize N- and P/Q-type channels, 15% only P/Q-type channels.
• In both groups of synapses release kinetics are determined by P/Q-type channels.
• We propose a more direct coupling of P/Q-type channels to synaptic release.

## Introduction

Transmitter release is tightly regulated by the presynaptic Ca^2+^ transient generated by voltage-gated Ca^2+^ channels (Ca_V_) (Katz and Miledi, 1965; Schneggenburger and Neher, 2005). In fact, synaptic vesicle fusion depends to the fourth to fifth power on the local Ca^2+^ concentration (Schneggenburger and Neher, 2000), which in turn is directly related to the number of presynaptic Ca_V_s (Schweizer et al., 2012). The three members of the high voltage-activated Ca_V_2 family, pharmacologically defined as P/Q- (Ca_V_2.1), N- (Ca_V_2.2) and R-type (Ca_V_2.3) channels, are the major presynaptic pore-forming subunits triggering synaptic release (Reid et al., 1998). In the majority of neurons of the central nervous system synaptic transmission depends cooperatively on both P/Q- and N-type (Olivera et al., 1994; Dunlap et al., 1995) channels. However, whether both channel types contribute to transmitter release in a synergistic manner or whether synapses utilize either N- or P/Q-type channels is a matter of intense and ongoing research (Reid et al., 2003).

In specific neuronal cell types or, even more precisely, synaptic connections presynaptic functions may depend on a single Ca_V_ type. For example transmitter release at the inhibitory basket cell–granule cell synapse in rat hippocampus is exclusively triggered by P/Q-type channels (Bucurenciu et al., 2010). However, for the majority of glutamatergic neurons three different scenarios have been reported. First, synapses can contain both channel types and synaptic transmission depends on the synergistic action of both channels. Indeed the application of selective N- and P/Q-type channel antagonists has revealed an additive contribution of both channels to the presynaptic Ca^2+^ signal (Wheeler et al., 1996; Wu et al., 1999; Reid et al., 2003) and thus transmitter release. Second, while hippocampal neurons express both channels at comparable levels (Schlick et al., 2010) individual synapses may differ in containing either N- or P/Q-type channels as the major presynaptic Ca_V_ type. This is supported by the observation that subpopulations of synapses are fully blocked by specific antagonists (Reuter, 1995; Reid et al., 2003). Finally, the third scenario assumes the coexistence of both channel types in synapses, yet their contribution to synaptic release may differ with respect to their coupling efficiency. This possibility is suggested by findings showing that in defined synapses neurotransmitter release was less sensitive to selective N-type inhibition when compared to P/Q-type inhibition, although the amount of N-type Ca^2+^ current was comparable (Qian and Noebels, 2001). Furthermore, analysis of evoked EPSC recordings in hippocampal neurons suggested the existence of P/Q-type preferring channel slots, which impose a ceiling on the synaptic efficacy (Cao et al., 2004). On the contrary, N-type channels clearly dominated when release was triggered by single action potentials and analyzed using fluorescent imaging of synaptic boutons (Ariel et al., 2012). Together this indicates a great variability in the contribution of presynaptic N- and P/Q-type channels to synaptic release. This heterogeneity of findings may originate from interactions of axons with specific postsynaptic targets determining presynaptic properties (Branco et al., 2008). Moreover, species differences between mouse and rat neurons and different experimental techniques and stimulation protocols used for the analysis of presynaptic function may also contribute to these inconsistencies.

Hence, to characterize the channel-type composition in nerve terminals of low-density cultured mouse hippocampal neurons, a frequently used model system (Kaech and Banker, 2006), we used quantitative analysis of FM dye destaining induced by sustained membrane depolarization (Hoopmann et al., 2012). Individual and combined application of the pharmacological blockers ω-agatoxin-IVA (Aga) and ω-conotoxin-GVIA (CTx), allowed us to specifically characterize the contribution of voltage-activated P/Q- and N-type channels, respectively. Our results suggest a more direct coupling of P/Q-type channels to synaptic release although the majority of synapses contained both N- and P/Q-type channels.

## Experimental procedures

### Ethical approval

Mice were bred and maintained at the central laboratory animal facility of the Medical University Innsbruck according to national and EU regulations and approved by the Austrian Science ministry (BM.W_F^a^). The number of animals used to obtain cells for this project was annually reported to the Austrian Science ministry (BM.W_F^a^).

### Cultured hippocampal neurons

Low-density cultures of hippocampal neurons were prepared from timed pregnant BALB/c mice on day 16.5 of gestation as previously described (Obermair et al., 2003, 2004, 2010; Kaech and Banker, 2006; Szabo et al., 2006). Briefly, pregnant mice were anesthetized in CO_2_, decapitated and the pups were removed from the uterus and decapitated. Isolated hippocampi were dissociated by trypsin treatment and trituration and plated on poly-l-lysine-coated glass coverslips in 60 mm culture dishes at a density of ∼5300 cells/cm^2^. After plating, cells were allowed to attach for 3–4 h before transferring the coverslips neuron-side-down into a 60 mm culture dish with a glial feeder layer. For maintenance neurons and glial feeder layer were cultured in serum-free Neurobasal medium (Invitrogen GmbH, Karlsruhe, Germany) supplemented with Glutamax and B27 supplements (Invitrogen GmbH, Karlsruhe, Germany). Ara-C (5 μM) was added 3 days after plating to stop the proliferation of non-neuronal cells.

### Imaging and analysis of FM dye release

**Fig. 1:**
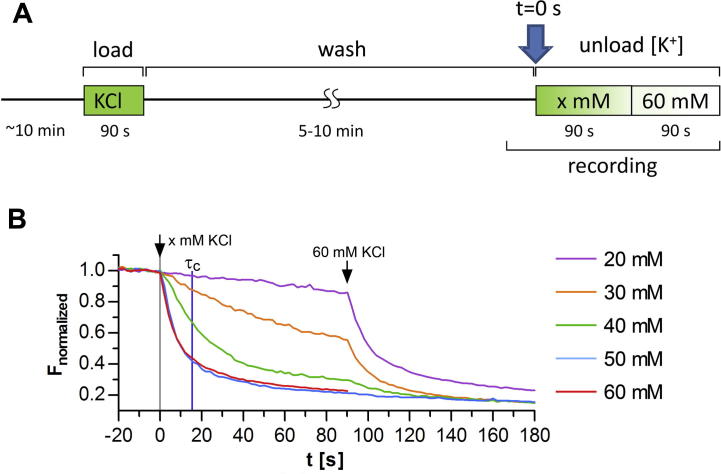
Dependence of FM dye release kinetics on the stimulus strength. (A) FM dye loading and unloading protocol. Unloading was initiated at *t* = 0 s by microperfusion of Tyrode solution with [K^+^] of 20, 30, 40, 50, or 60 mM. At the end of each recording cells were perfused with 60 mM [K^+^] to verify the release competence of synapses. (B) Normalized mean destaining curves (Δ*F*) of FM dye loaded synapses upon increasing [K^+^] concentrations. All synapses showed similar FM dye release at 60 mM [K^+^] (arrow). The blue vertical line marks the *τ* of the control condition (*τ*_c;_ 15.4 ± 8.9 s [mean ± SD]) at which fractional release amplitudes (*R*_f_; see Fig. 2) were determined. Curves represent mean values of 345, 199, 190, 316, 712 (for 20–60 mM) synapses from 5–18 experiments from 2–3 separate culture preparations.

18 mm cover glasses with cultured hippocampal neurons (days in vitro (DIV) 17–31) were mounted in a Ludin chamber in a modified tyrode solution (130 mM NaCl, 2.5 mM KCl, 2 mM CaCl_2_, 2 mM MgCl_2_, 10 mM HEPES, 30 mM glucose, 10 μM CNQX, 50 μM DL-AP5, pH 7.4 with NaOH). Distinct high KCl concentrations ([K^+^]) were generated by equimolar replacement of NaCl by KCl. Synaptic vesicles were loaded by bath perfusion of 60 mM [K^+^] Tyrode solution containing 15 μM FM1–43 for 90 s (Fig. 1A). After 5–10 min of continuous rinsing, synaptic release was induced by applying varying concentrations of [K^+^] through a microperfusion applicator for 90 s. After inducing FM dye release with varying [K^+^] the overall responsiveness of the synapses in all experiments was confirmed by a second depolarization step using 60 mM [K^+^], which resulted in a robust FM dye release independent of presynaptic N- and P/Q-type channels. Twelve-bit grayscale images were recorded at 0.5 Hz using an inverted Zeiss Axiovert 200 M epifluorescence microscope (Carl Zeiss Inc., Jena, Germany) equipped with a cooled CCD camera (SPOT; Diagnostic Instruments, Stirling Heights, MI, USA), Metavue image processing software (Universal Imaging, Corp., West Chester, PA, USA), and a 63 × 1.4 NA Zeiss Plan Apochromat oil immersion objective. Recordings were performed at 26 °C. For quantification of FM dye destaining, circular regions of interest (ROI, 3 × 3 pixels) were located manually over the center of FM dye fluorescence of spatially separated synapses. Their average intensity was measured using the ImageJ software package (Wayne Rasband, National Institutes of Health, Bethesda, MD, USA). The background-corrected intensity was normalized to the average intensity of the last five frames before initiation of high [K^+^] stimulation. The FM dye release curves of single synapses were fitted mono-exponentially from 2 to 90 s using Clampfit 10.2 (Molecular Devices, Downingtown, PA, USA). A successful mono-exponential fit was defined by positive values for amplitude (*A*), time constant (*τ*), and offset. Fits not fulfilling all criteria were excluded from further analyses. In order to obtain an additional and unbiased measure of release efficacy including all release traces (also the traces which did not fulfill the mono-exponential fitting criteria), we determined the fractional release (*R*_f_) of each synapse in each condition. *R*_f_ is defined as the percentage of total release determined at the time constant (*τ*) of the respective control condition. Standard errors are in the order of 1% and lower and thus not presented in the release diagrams.

### Drug and toxin application

Sham treatment with the equivalent amount of sterile MilliQ H_2_O was used for control. During FM release experiments 1 μM ω-conotoxin GVIA (CTx; Bachem, Bubendorf, Switzerland), or 0.2 μM Aga (Santa Cruz Biotechnology, Santa Cruz, CA, USA), or 1 mM CdCl_2_ (Sigma Aldrich, St. Louis, MO, USA) were applied after FM dye washout for 10 min before the experiment and were present in the locally applied tyrode and the high mM KCl solution.

### Statistical analysis and data presentation

Data were analyzed using Microsoft Office Excel (Microsoft Corporation, Redmond, WA, USA), GraphPad Prism (GraphPad Software Inc.; La Jolla, CA, USA), SPSS (IBM Corporation, Armonk, NY, USA), and SigmaStat (Systat Software, San Jose, CA, USA). Statistical significance was determined as indicated. *P*-values <0.05 were considered as statistically significant. If not otherwise noted graphs show medians, interquartile ranges (box) and 10th and 90th percentiles (whiskers); means are displayed as red triangles.

## Results

### Dependence of depolarization-induced FM dye release on the stimulus strength

Cultured hippocampal neurons serve as a widely used model system to study the properties of synaptic transmission using both electrophysiology (e.g. Tokuoka and Goda, 2008; Cao and Tsien, 2010) and functional fluorescent imaging of single synapses (e.g. Obermair et al., 2004, Branco et al., 2008). Activity-dependent unloading of fluorescent styryl dyes (FM dyes) from recycling synaptic vesicles provides a direct measure of synaptic function at the level of single synapses (Hoopmann et al., 2012). Here we used sustained membrane depolarization induced by varying extracellular K^+^ concentrations [K^+^] to trigger synaptic FM dye release. High [K^+^]-induced depolarization serves as a unique tool in that it basically voltage-clamps the neurons at different membrane potentials thus allowing the analysis of the contribution of Ca_V_s to synaptic FM dye release. We have previously demonstrated a strong developmental increase of Ca_V_2.1 α_1_ subunit mRNA expression in cultured hippocampal neurons (Schlick et al., 2010). Therefore, to avoid interference of developmental shifts in channel types with the analysis of the relative contributions to synaptic release we restricted our analyses to mature cultured neurons (>DIV17) in the present study.

While depolarization with up to 90 mM [K^+^] has frequently been used to activate pre- and postsynaptic Ca_V_s little information is available on the dependence of presynaptic release on the stimulus strength. Therefore to test the dependence of presynaptic FM dye release on the extent of [K^+^]-induced depolarization synaptic vesicles were loaded with FM dye by a 90 s bath perfusion using 60 mM [K^+^] Tyrode (Fig. 1A). Subsequently cells were continuously perfused for 5–10 min with 2.5 mM [K^+^] Tyrode in order to wash out excess FM dye from neuronal membranes. Synaptic FM dye release was induced by applying Tyrode solution with a defined [K^+^] through a microperfusion applicator positioned at a distance of approximately 100 μm from the respective neuron for 90 s. FM dye release triggered by the loading concentration of 60 mM [K^+^] could be blocked by the general Ca^2+^ channel blocker Cd^2+^ (1 mM) but it could neither be inhibited by antagonists of presynaptic Ca^2+^ channels (CTx or Aga) nor by the L-type channel blocker nifedipine (data not shown). Together this suggests that 60 mM [K^+^] induces a strong depolarization activating various independent voltage-activated and potentially secondary Ca^2+^ sources (e.g. Ca^2+^-induced Ca^2+^ release) resulting in non-specific neuronal Ca^2+^ signals leading to full FM dye release. Therefore we used 60 mM [K^+^] depolarization as FM dye-loading stimulus and to confirm the release competence of synapses after the application of graded [K^+^] concentrations (Fig. 1B).

To determine the optimal [K^+^] to analyze the contribution of presynaptic Ca_V_s to FM dye release, we triggered release with extracellular [K^+^] ranging from 20 to 60 mM (Fig. 1B). Depolarization with 20 mM [K^+^] did not induce FM dye release. Destaining of synaptic FM dye clusters, defined by a monoexponential loss of fluorescence, was first observed at [K^+^] of 30 mM and gradually increased up to 50 mM. At this concentration release curves reached the maximum capacity, as they could not be further enhanced by an additional increase to 60 mM.

### Properties of depolarization-induced FM dye release in cultured hippocampal neurons

The amplitude and the time course of synaptic FM dye release have been shown to provide direct readouts of the vesicle pool size and the release probability, respectively, at the level of single synapses (Ryan, 2001). Thus, in order to analyze release amplitudes (*A*) and time constants (*τ*), FM dye release traces of all individual synapses were fitted by a mono-exponential function (see Experimental procedures).

**Fig. 2:**
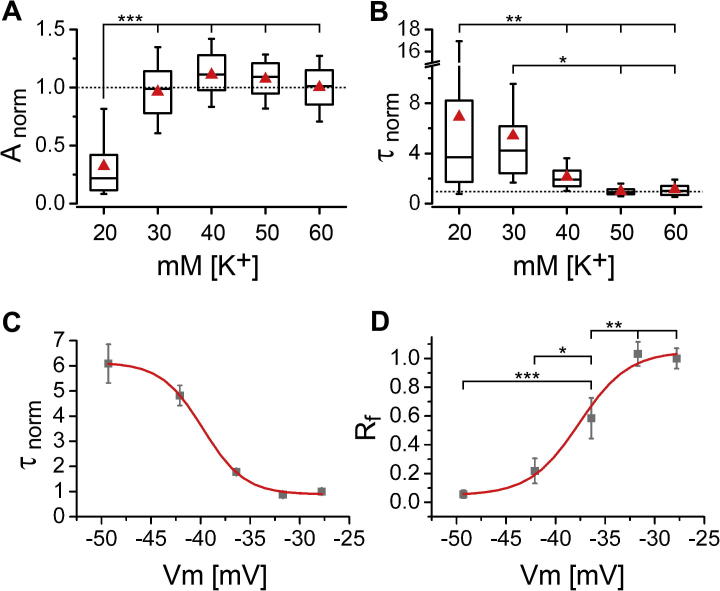
Characterization of depolarization-induced FM dye release. (A, B) FM dye release curves of all single synapses were fitted mono-exponentially. The monoexponential fitting parameters amplitude (*A*) and *τ* (B), were normalized to the mean and the median of the 60 mM [K^+^] condition, respectively, within each separate culture preparation. Box plots show median (horizontal line), interquartile range (box) and 10th and 90th percentiles (whiskers); mean is displayed as red triangle. (C) Boltzmann fit of normalized mean time constants (mean ± sem) plotted against the respective membrane potential as calculated by the Goldman–Hodgkin–Katz equation (half maximum activation = −39.8 mV). (D) Boltzmann fit of fractional release amplitudes (*R*_f_; mean ± sem) relative to the release observed at *τ* of the 60 mM [K^+^] condition (*τ*_c_, blue vertical line in Fig. 1B: 15.4 ± 8.9 s [mean ± SD]; half maximum activation = −37.6 mV). Data represent average values of 108, 113, 177, 313, 703 (20–60 mM; B, C) and 345, 199, 190, 316, 712 (20–60 mM; D) synapses. [ANOVA: *F*_(4, 40)_ = 16.1, *p* < 0.001 (A), *F*_(4, 40)_ = 3.7, *p* = 0.011 (B), *F*_(4, 40)_ = 29.2, *p* < 0.001 (D); Holm–Sidak post hoc test: ^∗^*p* < 0.05; ^∗∗^*p* < 0.01; ^∗∗∗^*p* < 0.001].

At all [K^+^] concentrations inducing synaptic release (30–60 mM) amplitudes were similar and significantly different from the 20 mM [K^+^] condition (Fig. 2A). This confirms that FM dye release at all [K^+^] concentrations depended on the same vesicle pool and that increasing the stimulus strength did not recruit additional pools within the observed time frame. In contrast, the rate of release sequentially increased (decreased time constants, *τ*) until saturation at 50 mM [K^+^] (Fig. 2B). This indicates that stronger depolarization of the membrane by increasing [K^+^] concentrations directly affected the release efficacy of the individual synapses. As evident in the mean release curves (see Fig. 1B) release was maximal at 50 mM [K^+^] and therefore time constants could not be further enhanced by stronger depolarization (Fig. 2B). Plotting the mean time constants against the corresponding membrane potential as calculated by the Goldman–Hodgkin–Katz equation (half maximum activation = −39.8 mV), revealed a sigmoid relationship of the release rate to the strength of depolarization (Fig. 2C).

One caveat in the analysis of the FM dye release components *τ* and amplitude is the exclusion of release curves not fulfilling the criteria set for mono-exponential fitting (see Experimental procedures). In this context it is important to note that the vast majority of synapses (i.e. release traces) not fulfilling these selection criteria showed either no release at all or an extremely slow and mostly linear release. The number of synapses with failed fits due to other (not immediately obvious) reasons is negligible. Therefore, for simplicity we refer to the entire population of synapses not fulfilling the selection criteria for mono-exponential fitting as non-responders. Excluding these non-responders introduces a strong bias in favor of strong depolarization by eliminating a large portion of synapses in the 20 and 30 mM [K^+^] condition from the analysis. In fact such non-responding synapses made up 69% and 43% of the synapses in the 20 and 30 mM conditions, respectively, whereas only 7% in the 40 mM and 1% each in the 50 and 60 mM condition were non-responding (Fig. 2, legend). Consequentially the *τ*’s at 20 and 30 mM are underestimates, which also explains why statistical analysis of these values did not show a significant difference from the 40 mM condition.

As an alternative and unbiased approach we introduced the analysis of the fractional release amplitude (*R*_f_). The fractional release amplitude (*R*_f_) represents the release amplitude in relation to the 100% release observed at the *τ* of the 60 mM [K^+^] control condition (mean *τ* ± SD: 15.4 ± 8.9 s), which is indicated in Fig. 1B by a blue vertical line. Thus *R*_f_ values could be determined from both responding and non-responding synapses. Using this approach all mean *R*_f_ values, except those between 20 and 30 mM as well as 50 and 60 mM [K^+^], were significantly different from each other (Fig. 2D). Like *τ* also mean *R*_f_ showed a pronounced sigmoid relationship with the membrane potential. Thus, in order to reliably identify potentially enhanced and reduced release properties by analyzing changes in *τ* (Fig. 2B) and in *R*_f_ (Fig. 2D), all subsequent experiments were performed with 40 mM [K^+^].

### Kinetics of high K^+^-induced synaptic release in cultured hippocampal neurons are determined by P/Q-type channels

**Fig. 3:**
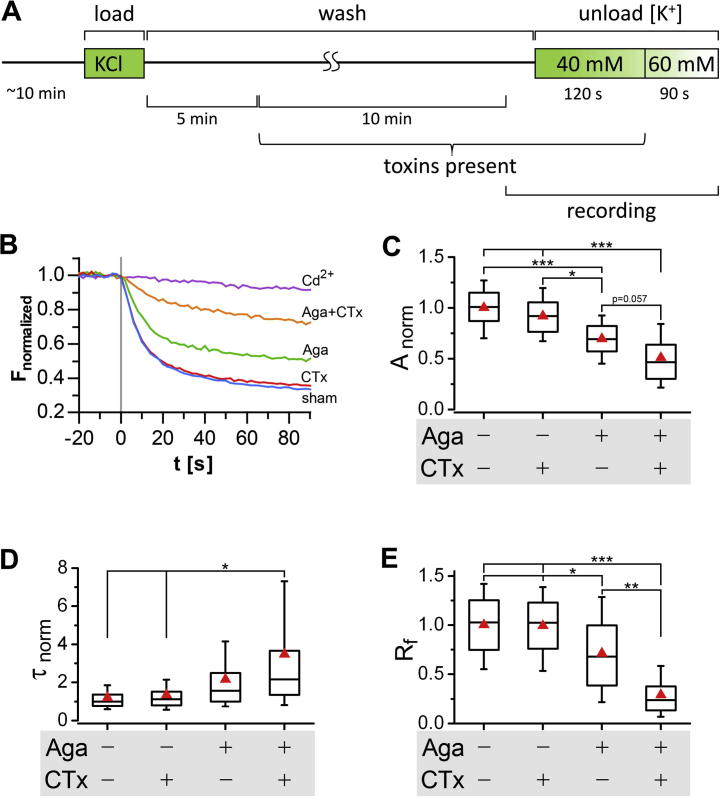
Pharmacological characterization of FM dye release reveals its prime dependence on P/Q-type channels. (A) FM dye loading and unloading protocol. Respective toxins were applied to the bath solution after a 5 min washing period and also applied through the microperfusion pipette during unloading at 40 mM [K^+^]. (B) Normalized FM dye release over time in the presence of different calcium channel blockers: Cd^2+^ (purple; *n* = 4 experiments/1 culture preparation/157 synapses), ω-agatoxin IVA (Aga; green; *n* = 12/3/474), ω-conotoxin GVIA (CTx; red; *n* = 14/3/556), sham (blue; *n* = 18/4/707), Aga + CTx (orange; *n* = 10/3/398). (C–E) The monoexponential fitting parameters and the fractional release (*R*_f_) derived from the release traces of individual synapses were normalized to mean amplitude (C), median *τ* (D), and mean *R*_f_ (E) of the parallel sham conditions. Box plots show median (horizontal line), interquartile range (box) and 10th and 90th percentiles (whiskers); mean is displayed as red triangle. Number of synapses (experiments) analyzed: 692 (18), 313 (14), 280 (12), 160 (10) (C, D, left to right) and 707 (18), 556 (14), 474 (12), 398 (10) (E) from 3 to 4 separate culture preparations. [ANOVA: *F*_(3, 50)_ = 12.8, *p* < 0.001 (C), *F*_(3, 50)_ = 2.8, *p* = 0.05 (D), *F*_(3, 50)_ = 12.0, *p* < 0.001 (E), Holm–Sidak post hoc test: ^∗^*p* < 0.05; ^∗∗^*p* < 0.01; ^∗∗∗^*p* < 0.001.]

Having established the basic properties of presynaptic FM dye release we next pharmacologically dissected the dependence of release at 40 mM [K^+^] on Ca^2+^ channels in order to reveal the contribution of the two major presynaptic channels P/Q- (Ca_V_2.1) and N-type (Ca_V_2.2). Dependence of FM dye release on Ca_V_s was first confirmed by applying the universal Ca^2+^ channel pore blocker cadmium (Cd^2+^; 1 mM), which completely abolished the loss of FM dye fluorescence (Fig. 3B). In order to test which types of presynaptic Ca^2+^ channels mediate [K^+^]-induced FM dye release we used individual or combined application of CTx and Aga at concentrations known to be highly specific for blocking N- and P/Q-type channels, respectively (Catterall et al., 2005). Incubation with CTx did not affect release kinetics and *R*_f_ (Fig. 3), suggesting that FM dye release induced by 40 mM [K^+^] does not critically depend on N-type channels. In contrast, treatment of cultures with Aga resulted in reduced amplitude and prolonged release kinetics. The combined application of CTx and Aga further reduced amplitude to approximately 50% of control and increased time constants more than 3-fold (Fig. 3C, D). These effects were most strikingly revealed by a reduction of the fractional release amplitude (*R*_f_) to 71% in Aga-only-treated neurons and to 29% in the combined CTx/Aga treatment. This synergistic effect of CTx and Aga indicates that the majority of synapses contain both N- and P/Q-type channels. Nevertheless, only P/Q-type channels can fully compensate the loss of N-type function, whereas N-type channels can compensate the loss of P/Q only with reduced efficacy.

### Quantitative analysis of the channel type contribution to 40 mM K^+^-induced FM dye release

**Fig. 4:**
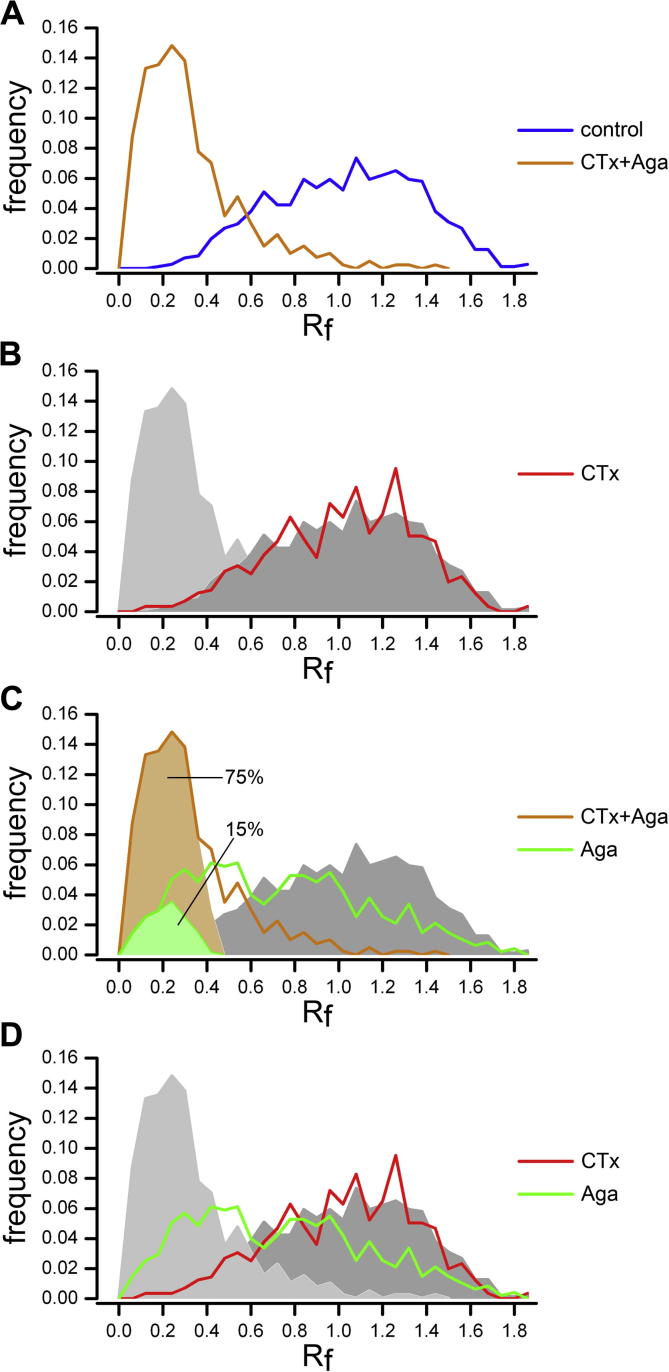
Dissecting the contribution of presynaptic Ca^2+^ channels to depolarization-induced FM dye release. Frequency distribution histograms of the *R*_f_ values of all synapses normalized to the mean of the control condition after pharmacological block of presynaptic Ca^2+^ channels. (A) Combined CTx and Aga application (orange) compared to control (blue). (B–D) For a better comparison of the CTx-only (red) and Aga-only (green) treatments the traces of the CTx–Aga and control treatments are displayed as light and dark gray shaded areas, respectively. See text for description. Number of synapses (experiments) analyzed: control, 707 (18); CTx, 556 (14); Aga, 474 (12); CTx + Aga, 398 (10) from 3 to 4 separate culture preparations.

In order to characterize the channel-type composition of individual synapses we generated frequency distribution histograms of *R*_f_ values of all individual recorded synapses in the different conditions (Fig. 4) normalized to the mean of the control. The *R*_f_ values of the control synapses show a wide normal distribution around the mean of 1.00 ± 0.33 (mean ± SD; Fig. 4A, blue line). In stark contrast, the distribution of the combined CTx and Aga block (*R*_f_ 0.29 ± 0.23; mean ± SD; Fig. 4A, orange line) displays a prominent peak at an *R*_f_ of approximately 0.2 – representing synapses with maximally blocked release – followed by a smaller population of synapses showing severely reduced release. Cluster analysis revealed that 75% of the synapses fall within this maximally blocked population (*R*_f_ 0.18 ± 0.10; mean ± SD; Fig. 4C, light orange area), indicating that release depended on presynaptic N- and P/Q-type channels. The tail of the skewed distribution represents the remaining 25% responding synapses, albeit at a strongly reduced efficacy. Comparing CTx-treated synapses with the control and combined CTx + Aga groups (Fig. 4B) reveals that the distribution of *R*_f_ values of CTx blocked synapses (red) is indistinguishable from the control condition (*R*_f_ 0.99 ± 0.32; mean ± SD). Thus blocking only N-type channels did neither affect the release properties of the entire synapse population, nor reveal a subpopulation of synapses entirely depended on N-type channels.

In contrast to blocking N-type channels, the *R*_f_ distribution of synapses treated with Aga only was clearly shifted in comparison with the control condition (Fig. 4C, green). This shift corresponds to an approximately 30% overall reduction in synaptic release (*R*_f_ 0.71 ± 0.40; mean ± SD; see also Fig. 3) and further shows that blocking P/Q-type channels affected release properties in the vast majority of synapses. The fact that in the Aga histogram a small population of synapses seems to group toward the lower, maximally blocked *R*_f_ values indicates that release in this subpopulation of synapses relies exclusively on P/Q-type channels. Indeed, by using a case-by-case exclusion approach we identified a 15% sub-population of fully blocked P/Q-only synapses (Fig. 4C, green shaded area) with a an *R*_f_ of 0.19 ± 0.09 (mean ± SD), which was not different from the identified cluster of maximally blocked synapses (orange shaded area; *t*-test: *t*_(367)_ = 0.169; *p* = 0.866).

While blocking N-type channels alone did not alter release properties the striking difference in the *R*_f_ distribution of the combined CTx + Aga block to the Aga-only block (Fig. 4D; light gray shaded area and green line, respectively) unmasks the presence of N-type channels in the majority of synapses. Because the synergistic CTx + Aga block shifted *R*_f_ values of the entire distribution toward maximal block we can conclude that, with the exception of the 15% P/Q-type-only synapses identified earlier (see above), 85% of the synapses indeed contain both, P/Q- and N-type channels. Finally, the difference in the *R*_f_ distributions between CTx-only (no effect) and Aga-only (shift of the entire population) treatments (Fig. 4D), clearly demonstrates that P/Q-type channels are more efficient in triggering depolarization-induced FM dye release.

Taken together we can show that ∼85% of the synapses contain both N- and P/Q-type and that in 15% of the synapses synaptic release depends exclusively on P/Q-type channels. Most importantly, the kinetics of synaptic release in synapses containing both channel types was determined by P/Q-type channels. Hence block of N-type channels can be entirely compensated by P/Q-type channels, whereas compensation of the P/Q-type block by N-type channels results in altered release kinetics.

## Discussion

N- and P/Q-type Ca_V_s are the main regulators of presynaptic transmitter release in neurons of the CNS, yet their distribution across synapses and their partially synergistic function is not fully understood. Here we used quantification of FM dye release induced by sustained membrane depolarization in cultured hippocampal neurons to characterize the contribution of N- and P/Q-type channels. Our results demonstrate that in 15% of synapses release depended exclusively on P/Q-type channels and 85% of synapses contain both N- and P/Q-type channels. However, kinetics of depolarization-induced FM dye release was determined by P/Q-type channels.

### Characteristics of K^+^-induced presynaptic FM dye release in cultured hippocampal neurons

Ca_V_s primarily trigger fast synchronous synaptic release, which is physiologically triggered by trains of action potentials (Neher and Sakaba, 2008; Gavello et al., 2012). Similar to bursts of action potentials high [K^+^] treatment results in sustained elevations of the presynaptic Ca^2+^ concentration. However, the duration and magnitude of high [K^+^]-induced Ca^2+^ transients is likely to exceed those of action potential bursts (Cohen and Segal, 2011). Therefore, during a long-lasting presynaptic Ca^2+^ transient the resulting FM dye destaining curve represents the balance of vesicle fusion and vesicle endocytosis and it is thus expected that the entire vesicle pool is depleted during a long depolarizing stimulus (Fernandez-Alfonso and Ryan, 2004; Cohen and Segal, 2011). Indeed, depolarization by 50 and 60 mM [K^+^], concentrations which have previously been used to analyze Ca^2+^ channel-dependent functions (Wheeler et al., 2012; Krey et al., 2013), resulted in a monoexponential decay and an 80% loss of total FM dye fluorescence after 90 s (Fig. 1B). However, release at these high concentrations, which depolarize the membrane to approximately −25 mV (Fig. 2C, D), resulted in a loss of specificity and apparently depended on various independent Ca^2+^ sources, as it was unaffected by the combined application the N- and P/Q-type channel blockers CTx and Aga. Titrating [K^+^] allowed us to determine conditions at which depolarization resulted in a graded response of FM dye release, both in terms of numbers of responsive synapses and in terms of the magnitude of release in individual synapses. 20 mM and 30 mM extracellular [K^+^], which depolarize the membrane to −50 and −42 mV, respectively (Fig. 2C, D), induced almost no (20 mM) and weak (30 mM) release. This was especially evident in the broad range of the release kinetics of the individual synapses (Fig. 2B). Moreover, 69% (20 mM) and 43% (30 mM) of the synapses were non-responding. This may suggest that the release observed in the subset of synapses in these conditions was triggered by a slightly increased open probability of the channels rather than a steady activation/inactivation state as expected for a stronger depolarization. In line with the explanation of minimal activation also the amplitude of FM dye destaining in 20 mM was significantly different from the other KCl concentrations.

By analyzing kinetics and *R*_f_ we could demonstrate that depolarization by 40 mM KCl (to approximately −35 mV) yielded synaptic release associated with FM dye destaining, which could also be statistically discriminated from the higher and lower depolarization strengths. Most importantly, the vast majority (75%) of release at 40 mM could reliably be blocked by the combined application of specific N- and P/Q-type Ca^2+^ channel blockers (Fig. 3E) and the remaining release showed severely reduced kinetics. The strongly reduced release kinetics suggests that this ∼20% remaining release may depend on distinct and slower calcium sources, for example, by different channel subtypes or by more distantly located calcium channels or both. In an attempt to dissect out a potential R-type channel contribution, which has been previously suggested (reviewed in Reid et al., 2003), we blocked Ca_V_s using 100 μM Cd^2+^ (data not shown). This experiment indeed showed a residual release comparable to the combined CTx and Aga block. Nevertheless, the reported IC50 of Cd^2+^ for R-type channels is 0.8 μM (Catterall et al., 2005) leaving the explanation of an R-type contribution inconclusive. Together our analysis demonstrates that synaptic release triggered by 40 mM KCl depolarization induces FM dye unloading with characteristic properties (kinetics and amplitude), which are dependent on presynaptic N- and P/Q-type Ca^2+^ channels.

### Role of presynaptic P/Q- and N-type channels in presynaptic FM dye release

**Fig. 5:**
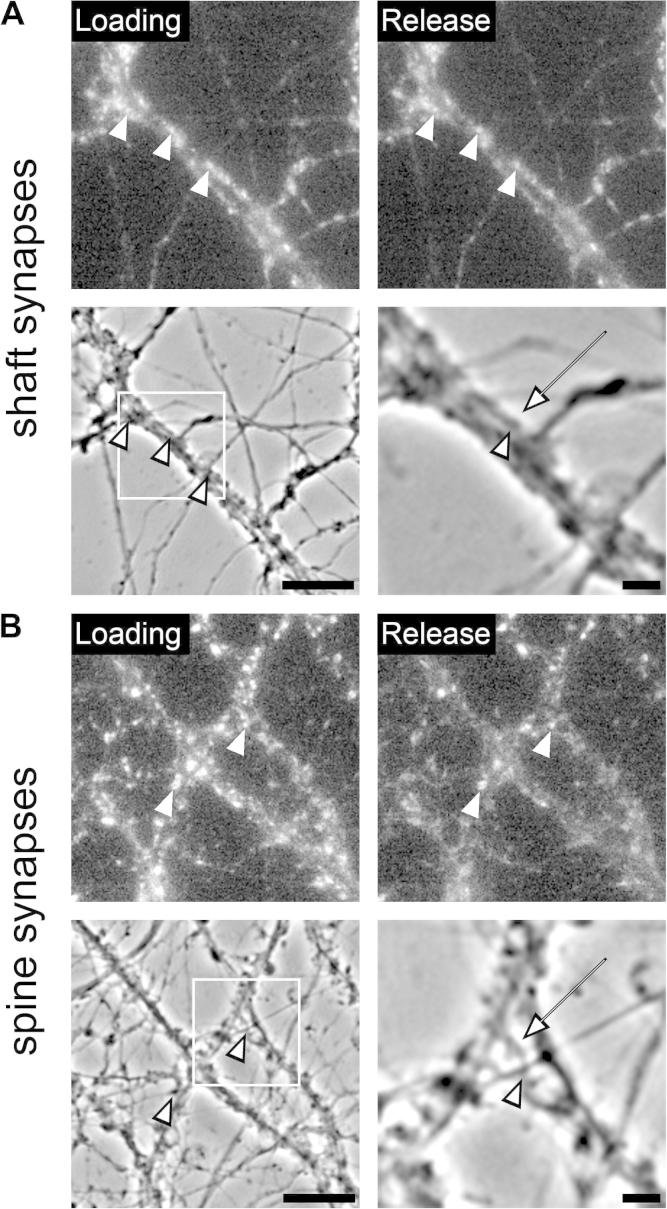
Localization of P/Q-type-only synapses on dendritic shafts and spines of hippocampal neurons. Following FM 1–43 dye experiments synapses in which release depended exclusively on P/Q-type channels were identified and located in the FM 1–43 dye images from before (Loading, upper panel) and during release (Release, upper panel) as well as in the corresponding phase contrast micrographs (lower panel). (A) Some of the synapses blocked by ω-agatoxin IVA (arrowheads) located on the dentritic shafts (arrow), a position typical for GABAergic synapses. (B) Synapses blocked by ω-agatoxin IVA that are positioned at a slight distance to the dendritic shaft (arrowheads). Magnification (lower panel, right) clearly shows such a synapse (arrowhead) terminating on the head of a dendritic spine (arrow), a position typical for glutamatergic synapses. Images were magnified using a bicubic interpolation to 400% and 1066% (magnified inset) of the original resolution. Bars, 5 μm and 1 μm (insets).

To understand which of the two types of presynaptic Ca^2+^ channels are involved in high potassium-mediated synaptic release in cultured hippocampal neurons, we characterized the individual or combined application of the N- and P/Q-type channel blockers CTx and Aga, respectively. If both channels are involved, we would expect release to be reduced according to the relative contribution of the channel (Wheeler et al., 1994). However, we identified that P/Q-type channels determined FM dye release kinetics, which is supported by three lines of evidence. First, single application of Aga shifted the release properties (*R*_f_) of the entire population of synapses (Fig. 4C, D). Second, blocking only N-type channels did not alter mean release kinetics and *R*_f_ (Fig. 3D, E). Third, Aga treatment alone induced a complete block of approximately 15% of synapses (Fig. 4D) while none of the synapses were blocked by CTx (Fig. 4B, D). The dependence of the release kinetics on P/Q-type channels is also in agreement with previous studies on neurotransmission in central glutamatergic neurons (Luebke et al., 1993; Kimura et al., 1995; Harvey et al., 1996; Ahmed and Siegelbaum, 2009). While kinetics was determined by P/Q-type channels, the majority of synapses also contained N-type channels. This is evident by the fact that the *R*_f_ value distribution of the entire synapse population is strongly left-shifted if CTx is applied together with Aga in comparison with the Aga-only condition (Fig. 4C). Thus, our study supports a model according to which the N- and P/Q-type channel distribution across the majority of synapses in cultured hippocampal neurons is non-uniform (Reid et al., 2003). However, it also revealed a small portion of synapses (15%) with segregated P/Q-type channel dependence. This segregated population could belong to a subset of randomly distributed synapses throughout all neurons, or it could indicate a preferential association of P/Q-type-only synapses with a special type of neuron. We have previously shown that cultured mouse hippocampal neurons consist of 90% glutamatergic and 10% GABAergic neurons (Obermair et al., 2003). Thus, a primary association of P/Q-type-only synapses with GABAergic neurons is feasible and has previously been reported (Bucurenciu et al., 2010). GABAergic synapses are typically located directly along the shafts of dendrites, while glutamatergic synapses are positioned at a slight distance to the dendritic shaft, preferentially terminating on dendritic spines (Obermair et al., 2003). Indeed, exemplary post hoc identification of synapses with P/Q-type channel-dependence revealed that some of these blocked synapses were located along the dendritic shaft, a localization indicative for GABAergic synapses (Fig. 5A). However, we also observed P/Q-only synapses being located juxtaposed to the dendritic shaft and terminating on dendritic spines (Fig. 5B), as is expected for glutamatergic synapses. Together this suggests the existence of a minor population of P/Q-channel-only synapses among both, GABAergic and glutamatergic synapses.

As mentioned above the dependence of release kinetics on P/Q-type channels is in agreement with previous studies. In our present study the analysis of depolarization induced FM dye release provides further mechanistic insight into the coupling of N- and P/Q-type channels to synaptic release. In line with the hypothesis on channel interacting slots (Cao and Tsien, 2010) blocking P/Q-type channels shifted release dependence to N-type channels. However, under this condition N-type channels triggered release at a markedly reduced efficiency (Fig. 3E and Fig. 4D). In the presence of Aga potential P/Q-type slots are still occupied by blocked P/Q-type channels and thus inaccessible for N-type channels. This suggests a more direct coupling of P/Q-type channels to the release site, likely by a closer association with the synaptic vesicle release machinery, similar to the situation at the Calyx of Held (Wu et al., 1999). Furthermore, differences in inactivation properties and G-protein-mediated inhibition of the two presynaptic Ca^2+^ channels may provide another explanation for the observed dominance of P/Q-type channels. Thus N-type channels show stronger inactivation at depolarized membrane potentials (Wykes et al., 2007), strong tonic inhibition by G proteins (Stephens et al., 1998), and less pronounced facilitation during bursts of action potentials (Currie and Fox, 2002). Finally FM dye kinetics during long-lasting depolarization may depend on the balance of exocytosis and endocytosis. Thus it is conceivable that the better efficacy in triggering FM dye release results from a stronger contribution of P/Q-type channels to both exo- and endocytosis. In this regard it may also reflect differences in the ability of presynaptic P/Q- and N-type channels to mediate asynchronous currents upon prolonged membrane depolarization (Few et al., 2012), which may contribute to the Ca^2+^ signal initiating presynaptic vesicle endocytosis (Yamashita, 2012).

## Conclusions

Taken together our data suggest a more direct coupling of P/Q-type over N-type channels to synaptic release. This dominance may explain the prevalence of channelopathies associated with Ca_V_2.1 (P/Q-type) such as familial hemiplegic migraine type 1, episodic ataxia type 2, and spinocerebellar ataxia type 6 (Pietrobon, 2010).

## Author contributions

G.J.O., B.E.F., and B.N. conceived the project plan. B.N. planned, performed, and analyzed FM dye experiments. G.J.O. and B.N. wrote the manuscript and all authors discussed, modified and approved the final version. G.J.O. initiated and supervised the project.
